# Evaluating the Effect of *Lacticaseibacillus paracasei* Strain Shirota on the Physical Consistency of Stool in Healthy Participants with Hard or Lumpy Stools: A Double-Blind, Randomized, Placebo-Controlled Study

**DOI:** 10.3390/nu16152469

**Published:** 2024-07-30

**Authors:** Satoshi Tsujibe, Agata Gawad, Akira Shigehisa, Kazunori Matsuda, Junji Fujimoto, Takuya Takahashi

**Affiliations:** 1 Yakult Central Institute, 5-11 Izumi, Kunitachi-shi 186-8650, Tokyo, Japan; akira-shigehisa@yakult.co.jp (A.S.); kazunori-matsuda@yakult.co.jp (K.M.); junji-fujimoto@yakult.co.jp (J.F.); 2 Yakult Honsha European Research Center for Microbiology VOF, Technologiepark 94, 9052 Ghent, East Flanders, Belgium; agata.gawad@yher.be (A.G.); takuya.takahashi@yher.be (T.T.)

**Keywords:** probiotics, stool-softening effect, *Lacticaseibacillus paracasei* strain Shirota, stool physical consistency, texture analyzer, Bristol stool form scale

## Abstract

We have earlier established a direct measurement method for assessing stool physical consistency using a texture analyzer (TAXT). The present study aimed to evaluate the stool softening effect of *Lacticaseibacillus paracasei* strain Shirota (LcS) using TAXT in a double-blind, randomized, placebo-controlled study. Sixty-four healthy participants with a Bristol stool form scale (BSFS) 1/2 ≥ 50% during screening consumed fermented milk containing LcS or a placebo beverage daily for 8 weeks. Stool consistency and water content were determined using TAXT and a lyophilizer, respectively. Participants evaluated their defecation using the BSFS. Stool consistency evaluated by a texture analyzer (TAXT) in the LcS group tended to be softer than that in the placebo group (*p* = 0.052). Subgroup analyses (TAXT value at baseline ≥ 4.5) showed that stool consistency was significantly softer in the LcS group (*p* = 0.014). Stool water content was also significantly higher in the LcS group than in the placebo group, but the proportion of normal stools was not statistically significant. We were unable to find evidence for the softening effect of LcS under the present study’s conditions. However, its efficacy may be confirmed by targeting participants with physically hard stools and TAXT values ≥ 4.5.

## 1. Introduction

Repeated defecation with constipated or diarrheal stools and irregular defecation (frequent or infrequent), observed in patients with functional constipation or irritable bowel syndrome, drastically reduces their quality of life [1]. Furthermore, several supposedly healthy individuals experience intestinal disorders, such as constipation or diarrhea, owing to an insufficient intake of dietary fiber [2,3,4,5], a lack of exercise [2,6,7,8], or chronic stress [9,10]. It has been reported that constipation is associated with cardiovascular disease [11,12,13] and colorectal cancer [14]; hence, regular bowel movements with normal stools are desirable to ensure good health. Stool consistency (i.e., stool hardness) is regarded as an important indicator of stool condition or bowel function.

The Bristol stool form scale (BSFS) is a 7-point Likert scale to visually evaluate stool form [15]. As the BSFS can be easily evaluated by participants themselves, the scale is widely used to estimate stool consistency. However, the BSFS is a surrogate and indirect method, and the results evaluated by participants are influenced by their sensations during/after defecation (e.g., straining, incompleteness) and inter-rater variability [16]. Therefore, the BSFS may not accurately evaluate stool consistency. Previously, we established a direct and objective measurement method of stool consistency using the TA.XTExpress Texture Analyzer (TAXT; Stable Micro Systems Ltd., Godalming, UK) [16]. In this study, it was confirmed that stool consistency measured by TAXT is strongly related to stool water content, and thus, the method can effectively evaluate stool consistency.

The human intestinal tract harbors approximately 40 trillion bacteria [17], and the commensal bacteria play a pivotal role in regulating the host’s physiological function [18]. The International Scientific Association for Probiotics and Prebiotics defines probiotics as “live microorganisms that, when administered in adequate amounts, confer a health benefit on the host” based on the definition by the Food and Agriculture Organization/World Health Organization [19,20]. Probiotic products contribute to regulating bowel function, including stool softening, in individuals with constipation [21,22]. Fermented milk containing *Lacticaseibacillus paracasei* strain Shirota (LcS; formerly *Lactobacillus casei* strain Shirota), a widely used probiotic strain, has a beneficial effect on bowel function [23,24,25,26,27]. Two research groups have reported that the daily intake of fermented milk containing LcS reduces the incidence of hard or lumpy stools [28,29]. However, these studies were not placebo-controlled, and stool consistency was evaluated solely based on the results from the self-reported BSFS. Consequently, the accuracy of evaluating the stool-softening effect of LcS remained unclear in these investigations.

Thus, we conducted a double-blind, randomized, placebo-controlled study to precisely assess the stool-softening effect of LcS using the TAXT instrument.

## 2. Materials and Methods

### 2.1. Study Design and Participants

This study was conducted at two sites in Belgium (Antwerpen and Mechelen) from June to November 2019 as a double-blind, randomized, placebo-controlled study. The participants were recruited from June to August 2019. In the 2-week screening period, participants themselves evaluated their defecations using the BSFS chart. Sixty-four healthy volunteers who frequently produced hard stools (BSFS 1 and 2 stools at a frequency of ≥50% during screening) were equally allocated to LcS or placebo groups. Participants in the LcS group consumed one bottle of a fermented milk beverage containing LcS daily for 8 weeks. Participants in the placebo group consumed one bottle of a placebo beverage (non-fermented milk without LcS) daily for 8 weeks. This study involved a stool collection period of 3 days at 3 timepoints (baseline, 4 weeks, and 8 weeks of intervention). The participants were instructed to collect every stool produced during the collection period and to record the BSFS score themselves for every defecation throughout this study. This study was registered in the ISRCTN registry (ISRCTN34762792).

### 2.2. Inclusion/Exclusion Criteria

The participants were included in this study if they met all of the following criteria:

- Informed consent obtained before engaging in any study-related activities.
- Healthy female or male aged 18–65 years (inclusive).
- Produced hard stool (BSFS 1 and 2 stools), with a frequency of 50% or more during the 2-week screening period.
- Did not take probiotics 2 weeks before the start of the screening period; if participants had used probiotics, a wash out period of 2 weeks was provided.
- Willing and able to collect every stool at home for 3 consecutive days, repeated three times during both the baseline and intervention period, store the samples in appropriate conditions, and return them within the specified timeframe.
- Willing and able to maintain a diary throughout the screening, baseline, and treatment period to collect information about the form of stools (based on the BSFS chart) and bowel habit.
- Committed not to change their current drinking, eating, smoking, and exercising habits during the course of the study.
- Proficient in understanding the Dutch or English language (reading, writing, and speaking).

The participants meeting any of the following criteria were excluded from study enrolment:

- A language barrier, mental or legal incapacity, unwillingness, or inability to understand or participate in the study.
- Vegetarian or vegan.
- Currently being treated, or treated within 1 month before screening, for constipation by a doctor.
- A history of gastrointestinal surgery, except appendectomy.
- A history of chronic/severe gastrointestinal disorders.
- Females of child-bearing potential who are pregnant, breast-feeding, intend to become pregnant, or were not using adequate contraceptive methods (e.g., oral contraceptive, condom, intrauterine device, abstinence, etc.).
- The inability to refrain from or the anticipation of antibiotic and/or laxative use.
- A history of drug and/or alcohol abuse.
- Milk allergies.
- Lactose intolerance.
- The presence of clinically significant disease which, in the investigators’ opinion, could compromise the safety of study participants or the study results.
- The use of prohibited concomitant medications (i.e., antibiotics, laxatives, herbal supplements or over-the-counter medications for diet attempts, anti-diarrhea medications, and anti-obesity medications).
- The use of disallowed concomitant products (i.e., prebiotic and probiotics products) within 2 weeks before the screening period, depending on whether a wash out period for probiotics was needed.
- Cancer (past or present, except basal cell skin cancer or squamous cell skin cancer) which, in the investigators’ opinion, could interfere with the results of the study.
- Previous participation in this study, with participation defined as screened; re-screening was not allowed.
- Participation in another interventional clinical study or receipt of any investigational product within 1 month before the screening period.

### 2.3. Test Beverages

The test beverage consisted of an LcS-containing beverage or a matching placebo beverage. LcS (strain no.: YIT 9029) was obtained from the Culture Collection Research Laboratory of Yakult Central Institute (Tokyo, Japan). One bottle (65 mL) of the test beverage contained 42.9 kcal/182 kJ, less than 0.1 g fat, 9.6 g carbohydrate, 0.01 g sodium, and 0.9 g protein. The LcS beverage was fermented milk containing at least 6.5 × 10^9^ colony-forming units of LcS per bottle. The ingredients were water, skimmed milk (reconstituted), glucose–fructose syrup, sugar, dextrin, flavoring agent, and LcS. The matching placebo beverage was non-fermented milk containing the same ingredients except LcS, and acidity was adjusted with lactic acid. These test beverages were produced at Yakult Europe B.V. (Almere, The Netherlands), distributed, and stored below 10 °C.

### 2.4. Sample Size

A comparable previous study was unavailable. In similar studies [23,24,28], 20–40 participants per group were enrolled to confirm the efficacy of LcS on bowel function, and therefore, we assumed that 30 participants per group were required to demonstrate the stool-softening effect of LcS. Assuming a study completion rate with no drop-out or serious protocol deviations of 0.95 (from our internal data), approximately 64 participants (32/group) were needed for this study.

### 2.5. Randomization

Randomization was performed by an independent statistician not involved in the study (Adriaens Consulting BVBA, Aalter, Belgium). Participant information regarding sex, age, and body mass index (BMI) collected at the sites were conveyed to the statistician. An equal distribution of variables such as sex, age, and BMI was ensured among the participants in each group. The unblinded statistician replaced the grouping information for each participant with a code (AAA or BBB) to maintain blinding for individuals not involved in the randomization. This blinded randomization list was then disseminated to the study sites, where the allocation was performed. Unblinding was carried out after database locking.

### 2.6. Ethics Statement

The ethics committee of SGS Life Sciences Clinical Pharmacology Unit Antwerpen (SGS) and AZ Sint-Maarten (AZSM) approved the study protocol (reference number: 5241 for SGS, EC 1928 for AZSM). All participants provided written informed consent. This study was conducted in compliance with the International Conference on Harmonization-Good Clinical Practice guidelines and applicable regulatory requirements, and in accordance with the most recent version of the Declaration of Helsinki.

### 2.7. Stool Specimens

A total of 494 whole stool specimens were collected from 64 participants. During the 3-day collection period, storage, and transportation to the laboratory, the stool specimens were maintained below 10 °C. The specimens were collected in a Commode Specimen Collection System (catalogue no. DYND36500; Medline Industries, Inc., Northfield, IL, USA) and stored in a portable refrigerator at the participants’ homes. Further, the specimens were submitted to the sites and transported to the laboratory. Processing and analysis were performed as soon as the specimens were delivered to the laboratory. All specimens were processed within 4 days after defecation. The measurement values of stool consistency remained constant during the 6 days of storage [16], and therefore, we used all of the specimens to measure stool consistency using TAXT.

### 2.8. Measurement of Stool Consistency

Stool consistency was measured using TAXT (Stable Micro Systems Ltd., Godalming, UK) with a 6 mm wide cylinder probe, according to a previous study [16]. Briefly, the whole stool was transferred into a plastic bag and manually homogenized for 30 s. A portion of the homogenized stool from each specimen was placed in a plastic container (cat. no. 75.562.105; Sarstedt AG & Co. KG, Nümbrecht, Germany) and incubated at 37 °C for 1 h. The gram force (g) against the probe was then measured five times at different points, and the average without the lowest and highest values was calculated. The measurement values were transformed to the natural logarithm (ln) and considered the stool consistency values (TAXT values, unit: ln g). The TAXT value was analyzed as the primary endpoint. For post hoc analysis, we calculated TAXT median values of the 3-day collection period for each participant.

### 2.9. Determination of Stool Water Content

Stool water content was determined using a lyophilizer. After homogenization, 4.5–5.5 g of stool was transferred to a plastic tube (cat. no. 80.734.001; Sarstedt AG & Co., KG) and weighed before freezing at −20 °C. After lyophilization, stool water content (%) was calculated based on the difference in weight, and the median value of the 3-day collection period for each participant was analyzed as one of the secondary endpoints.

### 2.10. BSFS Classification

The BSFS defines seven categories of stool form [15]. Participants selected the category that most closely resembled their stools at every defecation and recorded the score in an electric diary. The results were analyzed as one of the secondary endpoints. Additionally, the collected stool specimens were scored by laboratory experts.

### 2.11. Statistical Analyses

Stool consistency and water content were continuous variables showing normal distributions; hence, they were analyzed using the linear mixed-effects model (LMM). The participant-rated BSFS scores were converted into counts of normal (BSFS 3, 4, and 5) and abnormal stools (the remaining BSFS categories) every 2 weeks and were analyzed using a generalized LMM (GLMM) with a binomial family. Additionally, the expert-rated BSFS scores were analyzed using a cumulative link mixed model (CLMM). In the intention-to-treat (ITT) population, three participants did not provide stool specimens at the baseline. The missing baseline data were imputed by the ‘lme_imp’ function of R package ‘JointAI’. The statistical analyses were performed in the R statistical computing environment (version 3.6.2). The R package ‘lme4’ and ‘GLMMadaptive’ were used for the LMM and GLMM with a binomial family, respectively. The R package ‘ordinal’ was used for the CLMM.

In the primary endpoint analysis, the unaggregated and median aggregated TAXT values post-baseline were treated as response variables for a priori and post hoc analysis, respectively. In the secondary endpoint analysis, the median aggregated stool water content post-baseline was considered the response variable. For participant-rated BSFS scores, the matrix of normal and abnormal stools post-baseline was considered the response variable. In an additional analysis, the expert-rated BSFS scores post-baseline were used as the response variable.

In the above-mentioned model analyses, the post-baseline values of each endpoint were treated as response variables and the treatment group (LcS vs. placebo) was considered the fixed-effects variable to estimate the overall treatment effect (i.e., post-baseline mean difference between the groups) of LcS on each endpoint. To adjust for the influence of baseline values on the post-baseline values (i.e., response variable), the median aggregated baseline value of TAXT, stool water content, and expert-rated BSFS scores were included in the relevant models as fixed effects. For participant-rated BSFS scores, the proportion of normal stools at baseline was treated as the fixed effect. The following variables were also included as fixed effects in each model to adjust for the influence of these variables on the response variable: site (Antwerpen vs. Mechelen), period (4 weeks vs. 8 weeks), sex (female vs. male), age, and BMI. As for the four categorical variables (treatment group, period, site, and sex), “Placebo”, “4 weeks”, “Antwerpen”, and “Female” were reference levels. The participants’ information (i.e., participant ID) was treated as a random effect (random intercept). The models were fitted via restricted maximum likelihood. Two-sided 95% confidence intervals, two-sided *p*-values, fixed-effect estimates, and random-effect variances were calculated. *p*-values < 0.05 were considered statistically significant, and *p*-values < 0.1 were considered a statistical trend. These settings were predesigned before database locking.

## 3. Results

### 3.1. Study Design and Descriptive Characteristics

**Figure 1:**
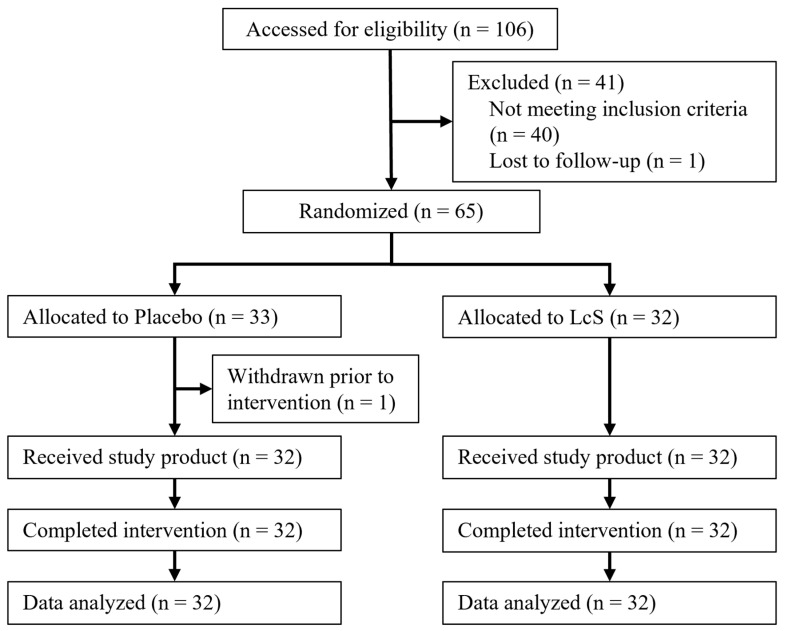
Study flowchart. LcS, *Lacticaseibacillus paracasei* strain Shirota.

Figure 1 shows the study flowchart. After assessing the eligibility of 106 participants, 65 were randomized and allocated to either the LcS or placebo group. One participant withdrew consent from participation in this study prior to intervention; sixty-four participants consumed the study products daily for 8 weeks and completed the study. All data collected from the 64 participants were used for statistical analyses and to evaluate the treatment effect in the ITT population, although six major protocol deviations (three were failure of stool collection at baseline and three involved the use of prohibited medications at baseline) were reported during the study. All adverse events (AEs) were reported throughout the present study, and an investigator at the study sites assessed the relationship between each AE and the intake of test beverages. Most AEs were deemed unlikely to be related to the intervention (Table S1), and therefore, the daily intake of test beverage for 8 weeks was considered safe.

The baseline characteristics of the 64 participants are summarized in Table 1. The distribution of age, BMI, sex, and participant number at each site was similar between the groups. The participant pool predominantly consisted of females in the present study (57/64), and this is consistent with previous findings revealing that constipation frequently occurs in females [30,31,32,33].

### 3.2. Stool Consistency Measured Using TAXT Instrument

**Figure 2:**
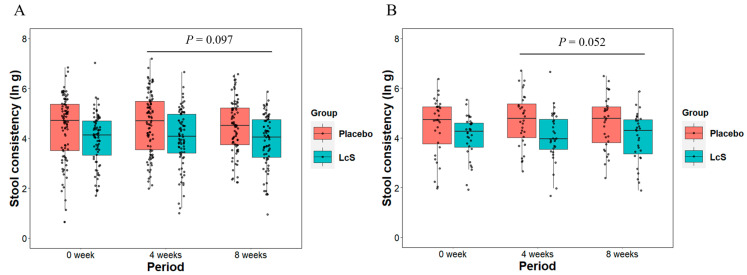
Changes in stool consistency measured using a texture analyzer (TAXT) throughout the study period. The boxplot shows (**A**) the unaggregated data in a priori analysis and (**B**) the median aggregated data in a post hoc analysis. The dots indicate individual TAXT data. Unit: natural log-transformed gram force (ln g). *p*-values: overall treatment effect between placebo and LcS groups (details in Table 2).

The boxplots in Figure 2 show the unaggregated and median aggregated TAXT values per group in each period. A priori analysis using the unaggregated TAXT results showed that the TAXT values post-baseline (4 weeks and 8 weeks) in the LcS group tended to be lower than those in the placebo group (treatment group variable in Table 2 (left), estimate = −0.33 [−0.71, 0.06], *p* = 0.097). The analysis indicated that the unexplained variance was considerably high [SD (within participant): 0.74 ln g, SD (between participants): 0.66 ln g in Table 2], and it can be considered that diet content or residuals of food material (e.g., food fiber) affect the variance of stool consistency. To restrain the large variance in TAXT values within participants, we conducted a post hoc analysis using the median aggregated TAXT values per participant in each period. The post hoc analysis showed that the post-baseline TAXT values in the LcS group tended to be lower than those in the placebo group (treatment group variable in Table 2 (right), estimate = −0.39 [−0.79, 0.00], *p* = 0.052).

### 3.3. Stool Water Content

**Figure 3:**
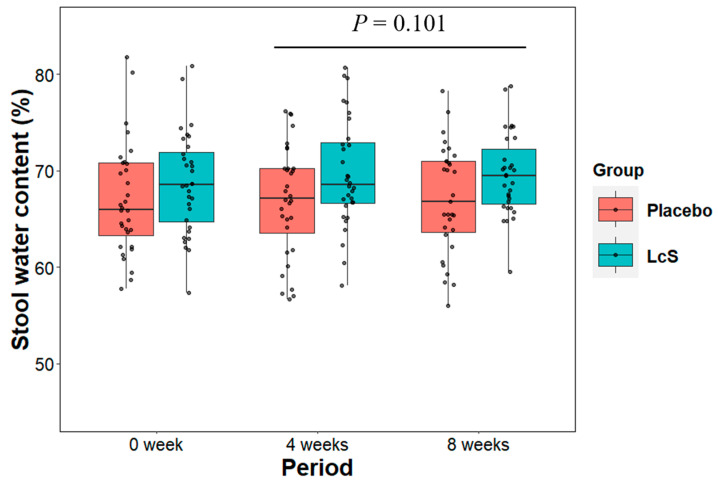
Changes in median aggregated stool water content throughout the study period. The dots indicate individual data of stool water content. *p*-values: overall treatment effect between placebo and LcS groups (details in Table 3).

Stool water content greatly impacts stool consistency. We investigated the effect of LcS intake on stool water content post-baseline. The boxplots in Figure 3 show the median values of stool water content per group for each period. The LMM analysis indicated that the difference between the two groups was not statistically significant (treatment group variable in Table 3 (left), estimate = 1.71 [−0.35, 3.77], *p* = 0.101).

### 3.4. BSFS Scores Evaluated by Participants and Experts

**Figure 4:**
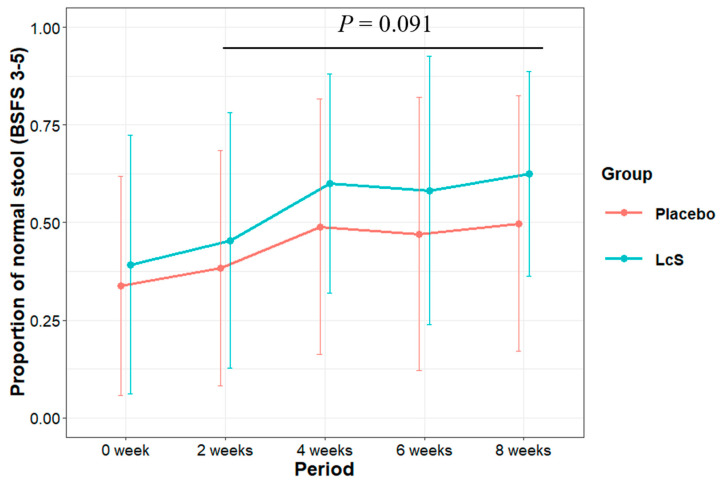
Changes in the proportion of normal stools throughout the study period. *p*-values: overall treatment effect between placebo and LcS groups (details in Table 3).

In the Rome IV criteria, BSFS 1 and 2 are defined as “constipation”; BSFS 6 and 7 are defined as “diarrhea” [34]. BSFS 3, 4, and 5 are defined as “normal” [9,10,35,36,37]. We categorized the participant-rated BSFS scores as normal (BSFS 3, 4, and 5) and abnormal stools (BSFS 1, 2, 6, and 7) and further analyzed the matrix of normal stools to abnormal stools using a GLMM with a binomial family. The line plots in Figure 4 show the changes in the mean value of the proportion of normal stools in the two groups. The GLMM analysis showed that the proportion of normal stools in the LcS group tended to be higher than that in the placebo group (treatment group variable in Table 3 (right), odds ratio (OR) = 1.62 [0.93, 2.84], *p* = 0.091).

We analyzed the expert-rated BSFS scores as an additional analysis. Unlike the analysis of the participant-rated BSFS scores, the expert-rated BSFS scores were limited to the collected stool specimens only and were analyzed using a CLMM. This analysis indicated that the likelihood of having higher BSFS scores tended to be higher in the LcS group than in the placebo group (treatment group variable in Table S2 (left), OR = 1.83 [0.94, 3.58], *p* = 0.076).

### 3.5. Subgroup Analysis Targeting the Participants Who Actually Produced Hard Stools at Baseline

Although we recruited participants who frequently produced hard stools based on the results of participant-rated BSFS scores in the screening period (BSFS 1/2 proportion ≥ 50%), many incidences of normal stools were observed at baseline in both groups (Table S3). Thus, we selected the participants who indeed produced hard stools and conducted an exploratory subgroup analysis. The majority of the specimens with BSFS scores of 1 and 2 exceeded a TAXT value of 4.5 (Table S4). The Rome IV criteria regard BSFS 1 and 2 as “hard stool” [34], and therefore, we defined a TAXT median value ≥ 4.5 at baseline as “actual hard stools” in the subgroup analysis.

**Figure 5:**
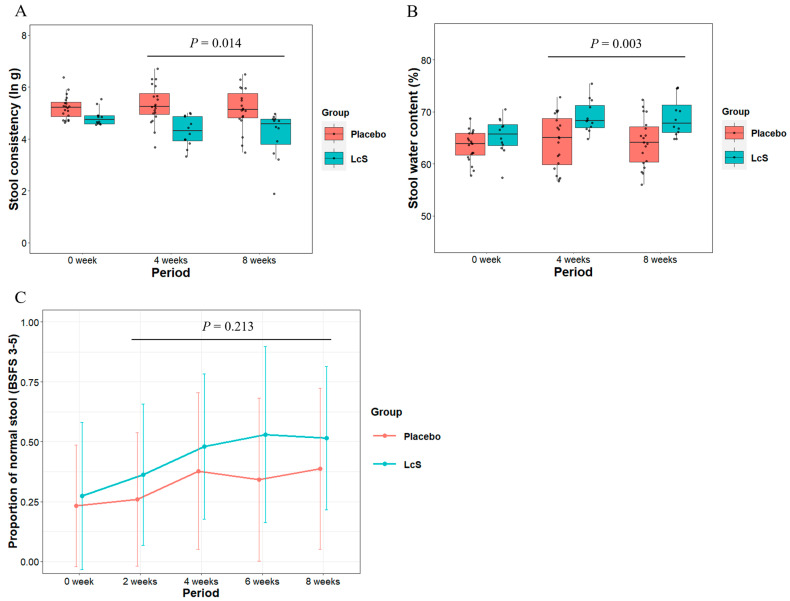
Changes in (**A**) stool consistency (unit: natural log-transformed gram force [ln g]), (**B**) stool water content, and (**C**) the proportion of normal stool in the hard stool subgroup (TAXT value at baseline ≥ 4.5 ln g). The dots indicate individual data. *p*-values: the overall treatment effect between placebo and LcS groups (details in Table 4).

The subgroup based on a baseline TAXT median value above 4.5 included 32 participants (Table S5; LcS group: 12, placebo group: 20). The subgroup analysis showed that the TAXT median value post-baseline in the LcS group was significantly lower than that in the placebo group (Figure 5A and treatment group variable in Table 4 (left), estimate = −0.74 [−1.31, −0.16], *p* = 0.014). Stool water content post-baseline was significantly higher in the LcS group than in the placebo group (Figure 5B and treatment group variable in Table 4 (middle), estimate = 4.83 [1.86, 7.81], *p* = 0.003). Moreover, the analysis of participant-rated BSFS scores showed that the mean value of the proportion of normal stools post-baseline was higher in the LcS group than in the placebo group (Figure 5C, treatment group variable in Table 4 (right), OR = 1.65 [0.75, 3.63], *p* = 0.213). The subgroup analysis of expert-rated BSFS scores showed that the likelihood of having higher BSFS scores tended to be higher in the LcS group than in the placebo group (treatment group variable in Table S2 (right), OR = 2.67 [0.91, 7.87], *p* = 0.074).

## 4. Discussion

Several studies have reported the effects of daily intake of fermented milk containing LcS on stool consistency [28,29]. However, these were not placebo-controlled studies, and stool consistency was evaluated using participant-rated BSFS scores. Thus, we conducted a double-blind, randomized, placebo-controlled study and employed the TAXT method to precisely evaluate the efficacy of LcS intake on stool consistency; to the best of our knowledge, this study is the first of its kind. The ITT analysis of TAXT values, stool water content, and BSFS scores showed that LcS intake may soften stools among healthy volunteers in Belgium. Although differences in TAXT values and stool water content were observed between the groups at baseline, these should be considered coincidental as the participants were selected based on the participant-rated BSFS category instead of the TAXT value or stool water content, and this study was randomized based on the information on sex, age, and BMI. Moreover, the post-baseline outcome was adjusted with the baseline outcome value, as prespecified in the statistical analysis plan. Therefore, the statistical results of the ITT analysis are considered reasonable. In addition, the subgroup analyses for the TAXT value and stool water content showed that in participants who actually produced hard stool and had a TAXT median value ≥ 4.5, the daily intake of LcS considerably softened stools. Although the results of the participant-rated/expert-rated BSFS analyses were not statistically significant, the ORs for the treatment group variable suggest that LcS intake may shift stool forms from harder (BSFS 1 or 2) to normal ones (BSFS 3–5). These findings suggest a potential beneficial effect of LcS on bowel function. However, we cannot unambiguously conclude that LcS has a stool-softening effect because it is unclear how the differences at baseline actually affect the estimate of treatment effect, and the subgroup analysis is likely biased due to the loss of randomization balance. Therefore, another randomized, placebo-controlled study targeting the participants with actual hard stools (e.g., TAXT value ≥ 4.5) is required.

Many participants with normal stools were enrolled in the present study despite selecting participants with a proportion of BSFS 1/2 ≥ 50% based on the results of participant-rated BSFS scores in the screening period. This unexpected scenario may most likely be the reason that we were unable to clearly identify the beneficial effect of LcS. Indeed, the results of participant-rated BSFS scores at baseline showed that 40 participants met the criterion of a proportion of BSFS 1 and 2 ≥ 50% (Table S5). There was a time lag of 1–2 months between the screening period and the baseline period due to the participants’ circumstances or study schedule, and therefore, the condition of some participants may have changed at the baseline period. Furthermore, expert-rated BSFS scores showed that only 28 participants met the above-mentioned criterion (Table S5). Although the BSFS has been broadly used to estimate stool consistency, subjectivity of the rater, particularly participants, probably affects the results of the BSFS. Indeed, Lemay et al. have reported that the distribution of technician-scored BSFS values is significantly different from that of self-reported BSFS scores [10]; we have also confirmed that participant-rated BSFS scores are affected by the participants’ sensation during/after their defecations [16]. Additionally, Chumpitazi et al. and Blake et al. have reported difficulties in distinguishing between BSFS 2 and 3 or 5 and 6 [36,37]. We also confirmed that there were multiple BSFS categories observed for a single stool in the present study; for example, the initial part of stool was observed to be BSFS 1–2, and the end part to be BSFS 3–4. These findings suggest that participants who are unfamiliar with the BSFS may be unable to categorize their stools accurately, and it may be inappropriate to use participant-rated BSFS scores for recruiting participants with hard stools. To ensure the recruitment of healthy volunteers with hard stools only, the time between the screening and baseline period should be as short as possible, and an objective method/indicator (e.g., TAXT value) instead of a subjective one (such as participant-rated BSFS score) should be employed.

During the subgroup analysis in the present study, we defined a TAXT value ≥ 4.5 as “hard stool”. The range (mean ± SD) of TAXT values corresponding to BSFS 2 and 3 was 5.042 ± 0.569 and 4.238 ± 0.681, respectively (Table S4). Assuming that the limit of the TAXT value between BSFS 2 and 3 was 4.5, the majority of stool samples with BSFS scores 1 and 2 exceeded this limit (96.9% and 84.1%, respectively). Our previous study has also shown that the majority of expert-rated BSFS scores of 1 and 2 exceed a TAXT value of 4.5 [16]. The assumption that a TAXT value of 4.5 is the limiting value between BSFS 2 and 3 is almost consistent with the Rome IV criteria. Therefore, our definition that a TAXT value ≥ 4.5 is regarded as “hard stool” can be considered valid.

As shown in Table S4, each BSFS category has a wide range of TAXT values. Even with expert-rated BSFS evaluations, 37.6% of specimens with BSFS 3, which is regarded as normal stool, exceeded a TAXT value of 4.5 and these stools should be regarded as hard stool. This finding indicates that there are limitations in evaluating stool consistency based on stool form alone. To our knowledge, this is the first study to evaluate the efficacy of probiotics on stool consistency using a direct measurement method. Since the TAXT method can provide objective and accurate results at high resolution, the TAXT results in the present study are more reliable than the BSFS results. However, the clinical significance of the TAXT value remains unclear. Stool consistency can be measured directly using the TAXT instrument [16,38], as well as a penetrometer [39,40,41,42,43], viscometer [44,45], or creepmeter [46]. These direct measurements have not been widely used because they require laboratory work, such as the handling/processing of stool specimens for measuring stool consistency. Therefore, data regarding physical stool consistency are still insufficient. Thus, the TAXT method needs to be further improved and simplified to achieve wide applicability in evaluating stool consistency. Further studies addressing physical stool consistency should also be conducted.

In summary, we could not verify that daily intake of fermented milk containing LcS softens hard stool, although the tendency toward stool softening was observed in the ITT analysis and statistically significant results were obtained in the subgroup analysis. Thus, this study’s findings suggest that the stool-softening effect of LcS may be elucidated by targeting participants with actual hard stools (TAXT median value ≥ 4.5 at baseline). Another double-blind, randomized, placebo-controlled study is required to validate these findings.

## Figures and Tables

**Table 1:** Participant characteristics at baseline. Age and BMI were analyzed using Student’s *t*-test. Sex and site were analyzed using chi-squared test. BMI, body mass index; SD, standard deviation.

		Statistic	Placebo (*N* = 32)	LcS (*N* = 32)	*p*-Value
Age (years)		Mean ± SD	47.3 ± 13.7	45.7 ± 13.2	0.65
		Median [lower/upper quartile]	50.5 [38.3, 59.0]	49.5 [36.8, 57.3]	
		Min, Max	18, 65	22, 62	
BMI (kg/m^2^)		Mean ± SD	24.6 ± 3.70	24.9 ± 3.95	0.79
		Median [lower/upper quartile]	24.2 [22.2, 26.2]	24.2 [22.5, 27.0]	
		Min, Max	19.1, 33.3	19.6, 38.9	
Sex	Female	*n* (%)	28 (87.5)	29 (90.6)	0.69
	Male	*n* (%)	4 (12.5)	3 (9.4)	
Site	Antwerpen	*n* (%)	17 (53.1)	19 (59.4)	0.61
	Mechelen	*n* (%)	15 (46.9)	13 (40.6)	

**Table 2:** Linear mixed-effects model (LMM) analysis of stool consistency (primary endpoint) in a priori analysis using unaggregated TAXT data (330 observations from 64 participants) and post hoc analysis using median aggregated TAXT data (126 observations from 64 participants).

Variables	Unaggregated TAXT Data (a Priori)			Median Aggregated TAXT Data (Post Hoc)		
	Estimates	CI	*p*	Estimates	CI	*p*
(Intercept)	2.51	0.78–4.24	0.005	2.44	0.74–4.15	0.006
Treatment group [LcS]	−0.33	−0.71–0.06	0.097	−0.39	−0.79–0.00	0.052
Baseline TAXT value	0.61	0.41–0.80	<0.001	0.60	0.41–0.80	<0.001
Period [8 weeks]	−0.07	−0.23–0.09	0.394	−0.07	−0.25–0.10	0.410
Site [Mechelen]	−0.25	−0.65–0.15	0.212	−0.21	−0.61–0.20	0.310
BMI	−0.02	−0.07–0.03	0.390	−0.02	−0.07–0.03	0.502
Age	0	−0.01–0.02	0.767	0	−0.01–0.02	0.816
Sex [Male]	−0.06	−0.68–0.56	0.840	0	−0.64–0.65	0.992
**Random Effects**						
SD (within participant)	0.74			0.49		
SD (between participants)	0.66			0.68		
Number of participants	64			64		
Observations	330			126		

The overall treatment effect (treatment group variable) was evaluated. Six fixed effects (baseline, period, site, BMI, age, sex) and one random effect (participant) were incorporated into the model for adjustment. As for the four categorical variables of treatment group (LcS vs. placebo), period (4 weeks vs. 8 weeks), site (Mechelen vs. Antwerpen), and sex (female vs. male), “placebo”, “4 weeks”, “Antwerpen”, and “female” were treated as references. *p* < 0.05 is considered statistically significant. TAXT, texture analyzer; BMI, body mass index; LcS, *Lacticaseibacillus paracasei* strain Shirota; CI, confidence interval; SD, standard deviation.

**Table 3:** Statistical analyses of secondary endpoints.

Variables	Stool Water Content			Ratio (Normal Stool/Abnormal Stool)		
	Estimates	CI	*p*	Odds Ratios	CI	*p*
(Intercept)	33.7	18.2–49.1	<0.001	0.20	0.02–2.10	0.180
Treatment group [LcS]	1.71	−0.35–3.77	0.101	1.62	0.93–2.84	0.091
Baseline data	0.51	0.32–0.70	<0.001	41.2	15.42–110.0	<0.001
Period [8 weeks]	−0.17	−1.30–0.96	0.765	1.13	1.09–1.18	<0.001
Site [Mechelen]	0.12	−2.01–2.26	0.909	1.09	0.60–1.97	0.783
BMI	0.03	−0.24–0.30	0.809	1.03	0.95–1.11	0.496
Age	−0.04	−0.12–0.04	0.303	0.97	0.95–0.99	0.015
Sex [Male]	−0.04	−3.42–3.34	0.981	0.82	0.32–2.07	0.673
**Random Effects**						
SD (within participant)	3.16			1.81		
SD (between participants)	3.35			1.05		
Number of participants	64			64		
Observations	126			2763		

Stool water content and participant-rated BSFS scores were analyzed using an LMM and generalized LMM with a binomial family, respectively. The overall treatment effect (treatment group variable) was evaluated. Six variables (baseline, period, site, BMI, age, sex) and one random effect (participant) were incorporated into the model for adjustment. As for the four categorical variables of treatment group (LcS vs. placebo), period (4 weeks vs. 8 weeks), site (Mechelen vs. Antwerpen), and sex (female vs. male), “placebo”, “4 weeks”, “Antwerpen”, and “female” were treated as references. *p* < 0.05 is considered statistically significant. TAXT, texture analyzer; BMI, body mass index; LcS, *Lacticaseibacillus paracasei* strain Shirota; SD, standard deviation; CI, confidence interval.

**Table 4:** Statistical analyses in hard stool subgroup (TAXT value at baseline ≥ 4.5 ln g).

Variables	Median Aggregated TAXT Data			Stool Water Content			Ratio (Normal Stool/Abnormal Stool)		
	Estimates	CI	*p*	Estimates	CI	*p*	Odds Ratios	CI	*p*
(Intercept)	2.25	−0.94–5.43	0.159	62.1	29.4–94.9	0.001	0.42	0.02–9.13	0.583
Treatment group [LcS]	−0.74	−1.31–−0.16	0.014	4.83	1.86–7.81	0.003	1.65	0.75–3.63	0.213
Baseline data	0.55	−0.12–1.21	0.102	0.13	−0.33–0.58	0.569	89.0	21.24–373.0	<0.001
Period [8 weeks]	−0.16	−0.40–0.08	0.189	−0.28	−2.03–1.47	0.744	1.18	1.11–1.26	<0.001
Site [Mechelen]	−0.12	−0.62–0.39	0.639	−1.51	−4.37–1.35	0.287	1.13	0.51–2.51	0.764
BMI	−0.03	−0.10–0.04	0.418	0.02	−0.38–0.41	0.927	0.98	0.88–1.10	0.774
Age	0.02	0.00–0.04	0.032	−0.12	−0.24–−0.01	0.029	0.96	0.93–0.99	0.020
Sex [Male]	−0.42	−1.18–0.34	0.268	1.50	−2.92–5.92	0.490	1.40	0.41–4.81	0.589
**Random Effects**									
SD (within participant)	0.46			4.24			1.81		
SD (between participants)	0.55			2.58			0.96		
Number of participants	32			32			32		
Observations	63			63			1347		

TAXT value and stool water content were analyzed using LMM. Participant-rated BSFS score was analyzed using GLMM. Overall treatment effect (treatment group variable) was evaluated. Six variables (baseline, period, site, BMI, age, sex) and one random variable (participant) were incorporated in model for adjustment. As for four categorical variables of treatment group (LcS vs. Placebo), period (4 weeks vs. 8 weeks), site (Mechelen vs. Antwerpen), and sex (female vs. male), “placebo”, “4 weeks”, “Antwerpen”, and “female” were treated as references. *p* < 0.05 is considered statistically significant.

## Data Availability

The data presented in this study are available on reasonable request from the corresponding author due to protection of participants’ privacy.

## Supplementary Materials

The following supporting information can be downloaded at: https://www.mdpi.com/article/10.3390/nu16152469/s1, Table S1: Adverse events (AEs) reported during study; Table S2: Cumulative link mixed model analysis of expert-rated BSFS scores; Table S3: Summary of participant- and expert-rated BSFS scores throughout study period in ITT population; Table S4: TAXT value corresponding to each BSFS category evaluated by expert; Table S5: Number of participants with hard stools at screening and baseline.
