# Supplementary material for: Evaluating the Effect of Lacticaseibacillus paracasei Strain Shirota on the Physical Consistency of Stool in Healthy Participants with Hard or Lumpy Stools: A Double-Blind, Randomized, Placebo-Controlled Study
Source: Nutrients. 2024 Jul 30;16(15):2469. doi: 10.3390/nu16152469 (PMC11313875; doi:10.3390/nu16152469)
Supplement: Supplementary file 1 [file nutrients-16-02469-s001.zip › nutrients-3104321-supplementary.pdf]

Evaluating the effect of *Lacticaseibacillus paracasei* strain Shirota on the physical consistency of stool in healthy participants with hard or lumpy stools: A double-blind, randomized, placebo-controlled study

Satoshi Tsujibe

Table S1

Adverse events (AE) reported during the study. LcS, *Lacticaseibacillus paracasei* strain Shirota.

Group	Reported term for the AE	Number of the observed AE	Relationship with the test beverage
**Placebo**	abdominal cramp	1	Unlikely
	backpain	2	Unlikely
	common cold	1	Unlikely
	concussion	1	Unlikely
	diarrhea	4	Unlikely
	dysmenorrhea	1	Unlikely
	flatulence	2	Unlikely
	headache	6	Unlikely
	nausea	1	Unlikely
	pain elbow	1	Unlikely
	rash from unknown origin	1	Unlikely
	stomach discomfort	3	Unlikely
	throat irritation	1	Unlikely
	urinary tract infection	1	Unlikely
	vomiting	1	Unlikely
**LcS**	backpain	1	Unlikely
	common cold	4	Unlikely
	conjunctivitis	1	Unlikely
	type 2 diabetes	1	Unlikely
	headache	3	Unlikely

	increased stool frequency and less hard stools	1	Possible
	migraine headache	1	Unlikely
	mood swings	1	Unlikely
	otitis media	1	Unlikely
	rhinitis	1	Unlikely
	sore throat	1	Unlikely
	stomach discomfort	3	Unlikely
	viral infection upper respiratory tract	1	Unlikely
	vomiting	1	Unlikely

Table S2

Cumulative link mixed model analysis of expert-BSFS. The overall treatment effect (treatment group variable) was evaluated. Six variables (baseline, timepoint, site, BMI, age, sex) and one random variable (participant) were incorporated into the model for adjustment. As for four categorical variables of the treatment group (LcS vs placebo), period (4 weeks vs 8 weeks), site (Mechelen vs Antwerpen), and sex (female vs male), "placebo", "4 weeks", "Antwerpen", and "female" were treated as references. $P < 0.05$ is considered statistically significant. ITT, intention-to-treat; TAXT, texture analyzer; BSFS, Bristol stool form scale; BMI, body mass index; LcS, *Lacticaseibacillus paracasei* strain Shirota; SD, standard deviation; CI, confidence interval.

	Expert-BSFS (ITT population)			***Expert-BSFS (Subgroup with TAXT value >4.5)***		
Predictors	***Odds Ratios***	***CI***	***P***	***Odds Ratios***	***CI***	***P***
1|2	2.47	2.46 – 2.48	<0.001	0.28	0.00 – 16.17	0.54
2|3	14.09	9.70 – 20.46	<0.001	1.89	0.03 – 107.38	0.758
3|4	246.93	148.42 – 410.83	<0.001	39.37	0.68 – 2285.47	0.076
4|5	2006.81	1044.54 – 3855.57	<0.001	279.8	4.21 – 18579.08	0.008
Treatment group [LcS]	1.83	0.94 – 3.58	0.076	2.67	0.91 – 7.87	0.074
Baseline BSFS	3.59	3.58 – 3.60	<0.001	3.54	1.47 – 8.51	0.005

Period [8 weeks]	0.99	0.98 – 0.99	<0.001	1.12	0.62 – 2.03	0.712
Site [Mechelen]	0.85	0.45 – 1.63	0.631	1.46	0.59 – 3.57	0.412
BMI	1.01	1.01 – 1.01	<0.001	0.96	0.85 – 1.09	0.539
Age	1	1.00 – 1.00	0.279	0.96	0.93 – 1.00	0.054
Sex [Male]	1.02	0.33 – 3.12	0.97	3.68	0.94 – 14.46	0.062
**Random Effects**						
SD (within participant)	1.81			1.81		
SD (between subject)	1.19			0.81		
Number of participants	64			32		
Observations	326			168		

Table S3

Summary of participant- and expert-BSFS scores throughout the study period in the ITT population. BSFS scores for each participant were median-aggregated by each period. Upper: participant-BSFS, Lower: expert-BSFS. ITT, intention-to-treat; BSFS, Bristol stool form scale; LcS: *Lacticaseibacillus paracasei* strain Shirota.

	**Placebo**										**LcS**									
	**0 week**		**2 weeks**		**4 weeks**		**6 weeks**		**8 weeks**		**0 week**		**2 weeks**		**4 weeks**		**6 weeks**		**8 weeks**	
**BSFS**	**number**	**%**	**number**	**%**	**number**	**%**	**number**	**%**	**number**	**%**	**number**	**%**	**number**	**%**	**number**	**%**	**number**	**%**	**number**	**%**
**1**	8	25	8	25	6	18.75	5	15.63	6	18.75	3	9.38	5	15.63	2	6.25	3	9.38	2	6.25
**2**	12	37.5	10	31.25	10	31.25	7	21.88	8	25	15	46.88	11	34.38	9	28.13	11	34.38	8	25
**3**	10	31.25	12	37.5	11	34.38	15	46.88	12	37.5	14	43.75	9	28.13	16	50	10	31.25	12	37.5
**4**	2	6.25	1	3.13	4	12.5	4	12.5	4	12.5	0	0	7	21.88	4	12.5	7	21.88	8	25
**5**	0	0	1	3.13	1	3.13	1	3.13	2	6.25	0	0	0	0	1	3.13	1	3.13	2	6.25
**6**	0	0	0	0	0	0	0	0	0	0	0	0	0	0	0	0	0	0	0	0
**7**	0	0	0	0	0	0	0	0	0	0	0	0	0	0	0	0	0	0	0	0
**Total**	32	100	32	100	32	100	32	100	32	100	32	100	32	100	32	100	32	100	32	100

	**Placebo**						**LcS**					
	**0 week**		**4 weeks**		**8 weeks**		**0 week**		**4 weeks**		**8 weeks**	
**BSFS**	**number**	**%**	**number**	**%**	**number**	**%**	**number**	**%**	**number**	**%**	**number**	**%**
**1**	7	21.88	3	9.38	4	12.9	1	3.13	2	6.25	0	0
**2**	12	37.5	11	34.38	12	38.71	9	28.13	4	12.5	3	9.68
**3**	6	18.75	13	40.63	10	32.26	10	31.25	14	43.75	18	58.06
**4**	2	6.25	3	9.38	4	12.9	11	34.38	10	31.25	7	22.58
**5**	5	15.63	2	6.25	1	3.23	1	3.13	2	6.25	3	9.68
**6**	0	0	0	0	0	0	0	0	0	0	0	0
**7**	0	0	0	0	0	0	0	0	0	0	0	0
**Total**	32	100	32	100	31	100	32	100	32	100	31	100

Table S4

TAXT value corresponding to each BSFS category evaluated by the expert. TAXT, texture analyzer; BSFS, Bristol stool form scale; SD, standard deviation.

**Expert-BSFS**		**Number of stool sample**	**TAXT value (ln g)**			**TAXT ≥4.5**	
			**Mean**	**SD**	**Median**	**Number**	**%**
**Hard stool**	**1**	65	5.731	0.679	5.743	63	96.9
	**2**	107	5.042	0.569	5.049	90	84.1
**Normal stool**	**3**	178	4.238	0.681	4.282	67	37.6
	**4**	99	3.368	0.709	3.461	3	3
	**5**	41	2.377	0.583	2.391	0	0
**Soft stool**	**6**	6	1.162	0.613	0.97	0	0
	**7**	1	-0.124	n.a.	-0.124	0	0

Evaluating the effect of *Lacticaseibacillus paracasei* strain Shirota on the physical consistency of stool in healthy participants with hard or lumpy stools: A double-blind, randomized, placebo-controlled study

Satoshi Tsujibe

Table S5

Number of participants with hard stools at screening and baseline. TAXT, texture analyzer; BSFS, Bristol stool form scale; LcS: *Lacticaseibacillus paracasei* strain Shirota.

		**LcS**	**Placebo**	**Total**
**Screening period**	**Participant-BSFS 1/2 ≥50%**	32	32	64 (100%)
**Baseline period**	**Participant-BSFS 1/2 ≥50%**	18	22	40 (62.5%)
	**Expert-BSFS 1/2 ≥50%**	10	18	28 (43.75%)
	**TAXT median value ≥4.5**	12	20	32 (50.00%)
